# Economics of alternative dosing strategies for pembrolizumab and nivolumab at a single academic cancer center

**DOI:** 10.1002/cam4.2888

**Published:** 2020-01-28

**Authors:** Evan Hall, Jenny Zhang, Eun Jeong Kim, Grace Hwang, Gilbert Chu, Shailender Bhatia, Sunil Reddy

**Affiliations:** ^1^ Division of Oncology Department of Medicine University of Washington Seattle WA USA; ^2^ Clinical Research Division Fred Hutchinson Cancer Research Center Seattle WA USA; ^3^ Department of Pharmacy Stanford University Stanford CA USA; ^4^ Stanford Healthcare Stanford CA USA; ^5^ Division of Oncology Department of Medicine Stanford University Stanford CA USA; ^6^ Department of Biochemistry Stanford University Stanford CA USA

**Keywords:** dosing strategy, health economics, neoplasms, nivolumab, pembrolizumab

## Abstract

**Background:**

The FDA initially approved pembrolizumab and nivolumab for doses based on patient weight, but subsequently amended approval to fixed doses. We estimated savings from novel dosing strategies based on real‐world patient data from a single cancer center.

**Methods:**

We analyzed all outpatient doses of pembrolizumab and nivolumab administered at three infusion centers affiliated with our academic hospital between July 1, 2018 and Oct 31, 2018. We estimated savings from several dosing strategies with and without vial sharing between patients.

**Results:**

A total of 1029 doses of pembrolizumab or nivolumab were administered for multiple cancer types. For 77% of doses, the weight‐based dose was less than the fixed dose. “Dose‐minimization” (DM), defined as the lesser of weight‐based and fixed dose decreased nivolumab spending by 9% without affecting pembrolizumab spending. DM *plus* vial sharing decreased pembrolizumab spending by 19% without affecting nivolumab. The differences in savings were due to availability of multiple vial sizes for nivolumab but not pembrolizumab. DM plus vial sharing for both drugs would have saved $1.5 million USD over the 4‐month study period.

**Conclusion:**

New dosing strategies for pembrolizumab and nivolumab can generate large savings without anticipated decrease in efficacy. Barriers include FDA dosing labels, hospital policies against vial sharing, and inaccessibility of smaller vial sizes, which are currently available in other worldwide markets.

## Graphical abstract

New dosing strategies of PD‐1 inhibitors can generate large cost savings without anticipated impact on anticancer efficacy. These strategies can be easily implemented at cancer centers and do not require cooperation from payers or pharmaceutical companies.

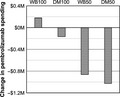

## 1 BACKGROUND

Immune checkpoint inhibitors are used for an increasing number of cancer types. Currently, nearly 50% of patients with advanced cancer are eligible for such drugs at some point during their treatment.^1^ This percentage will likely increase with results from the more than 2000 CPI clinical trials currently underway.^2^ Immune checkpoint inhibitors nivolumab and pembrolizumab were the second and third highest selling cancer drugs in 2018, accounting for $14 billion USD in spending.^3^

Initial clinical trials used weight‐based doses for pembrolizumab and nivolumab for metastatic melanoma and non‐small cell lung cancer.^4^, ^5^, ^6^, ^7^, ^8^, ^9^, ^10^, ^11^ Based on metastatic melanoma trials, the FDA approved pembrolizumab at a dose of 2 mg/kg every 3 weeks in September 2014, and nivolumab at a dose of 3 mg/kg every 2 weeks in October 2014. Subsequently, the FDA approved both drugs for non‐small cell lung cancer with the same weight‐based doses.

In February 2018, the FDA modified weight‐based doses of nivolumab and pembrolizumab given as monotherapy to fixed doses (200 mg every 3 weeks for pembrolizumab; 240 mg every 2 weeks for nivolumab). The changes were propelled by efficacy of fixed doses in several clinical settings and on pharmacokinetic data showing similar patient exposure for weight‐based and *appropriate* fixed doses.^12^, ^13^, ^14^, ^15^, ^16^, ^17^

Several studies have used models to show that changing from weight‐based to fixed doses increases spending. In one study, first‐line pembrolizumab spending in the US for non‐small cell lung cancer increased by more than $800 million USD annually.^18^ In another study, pembrolizumab and nivolumab spending in France increased by €55 million, or $61 million USD annually.^19^

Clinical studies have failed to detect differences in efficacy among the approved fixed doses and various weight‐based doses. Therefore, we estimated potential savings from different dosing strategies. Unlike the previous studies, data from our single institution allowed calculations based on all of the following key elements of patient data: actual patient weights, daily infusion center patient volumes, and physician prescribing practices.

## 2 METHODS

### 2.1 Checkpoint inhibitor utilization data

With approval of the Institutional Review Board from our academic medical center, we used an institutional database to retrospectively identify all outpatient doses of pembrolizumab and nivolumab given at three infusion centers affiliated with our center between July 1, 2018 and Oct 31, 2018. Demographic data included cancer type, treatment date, treatment site, and patient weight. We excluded doses of nivolumab given concurrently with ipilimumab, because weight‐based doses are standard in this setting. We also excluded doses administered: (a) without patient weight information; (b) without adherence to either weight‐based or fixed dosing; and (c) within a clinical trial. We studied doses administered as either a fixed dose (pembrolizumab 200 mg every 3 weeks; nivolumab 240 mg every 2 weeks or 480 mg every 4 weeks), or a weight‐based dose (pembrolizumab 2 mg/kg every 3 weeks; nivolumab 3 mg/kg every 2 weeks or 6 mg/kg every 4 weeks). To calculate actual utilization of pembrolizumab and nivolumab, we accounted for the entire contents of each opened vial, including contents not infused into the patient.

### 2.2 Economic modeling

#### 2.2.1 Weight‐based dosing with and without vial sharing

Weight‐based doses were calculated from patient weights documented in the clinical database. We modeled the impact of universal weight‐based dosing under two conditions: with and without "vial sharing." Under the model for vial sharing, the drug remaining from a vial opened for one patient could be used for subsequent patients treated at the same site on the same day. The calculation of drug utilization included drug remaining in vials at the end of the day. We modeled alternative dosing strategies on a day‐ to‐day, site‐ to‐site analysis using drug vial sizes currently available in the US (pembrolizumab 100 mg vials only; nivolumab 40, 100, and 240 mg vials).

#### 2.2.2 Dose minimization

We modeled a novel “dose‐minimization” strategy, defined as using the lesser of the weight‐based and fixed dose for each patient. In other words, dose minimization would use the weight‐based dose, employing drug vial sizes available in the US, but capping the allowed dose at the fixed dose and accounting for drug left over at the end of each treatment day.

### 2.3 Drug pricing estimates

Estimates used the average sales price (ASP) from Center for Medicare and Medicaid Services for Part B drugs: $47.35 USD per mg for pembrolizumab, and $27.54 USD per mg for nivolumab.^20^

## 3 RESULTS

A total of 1110 doses of pembrolizumab and nivolumab were administered over the 4‐month study period. We analyzed 1029 doses, representing 271 unique patients across multiple cancer types. The 81 doses excluded from analysis included 50 doses in clinical trials, 24 atypical doses, and 7 doses without a concurrent patient weight. Nearly all doses (94%) were administered as fixed doses.

We collected data for drug doses, patient weights, and tumor types (Table 1). Mean patient weight was 73.3 kg for pembrolizumab, 81.9 kg for nivolumab, and 76.3 kg overall, with weights ranging from 38 to 175 kg. The weight‐based dose was less than the fixed dose for 90% of pembrolizumab doses and 53% of nivolumab doses, corresponding to 77% of all doses.

**Table 1:** Drug doses and tumor types

	Pembrolizumab	Nivolumab	Total
Doses and weights			
Fixed dose	200 mg	240 mg	NA
Weight‐based dose	2 mg/kg Q3W	3 mg/kg Q2W or 6 mg/kg Q4W	NA
Patient mean weight	73.3 kg	81.9 kg	76.3 kg
Patient median weight (range)	69.9 kg (38‐118 kg)	78.4 kg (41‐175 kg)	74.9 kg (38‐175 kg)
Total doses	665 (100%)	364 (100%)	1,029 (100%)
Fixed doses	620 (93%)	351 (96%)	971 (94%)
Weight‐based less than fixed dose	596 (90%)	193 (53%)	789 (77%)
Tumor type			
Lung	260 (39%)	35 (10%)	295 (29%)
Melanoma	119 (18%)	75 (21%)	194 (19%)
Urothelial/bladder	44 (7%)	0 (0%)	44 (4%)
Head/Neck	39 (6%)	92 (25%)	131 (13%)
Liver	0 (0%)	52 (14%)	52 (5%)
Kidney	11 (2%)	51 (14%)	62 (6%)
Other	192 (29%)	59 (16%)	251 (24%)

Abbreviations: %, percent of doses relative to total doses; NA, not applicable.

Modeling a transition from our existing practice to universal weight‐based doses increased spending by 2.4% for pembrolizumab and 4.2% for nivolumab (Table 2). The transition to universal weight‐based doses *plus* vial sharing increased spending by 2.9% for nivolumab, but decreased spending by 18% for pembrolizumab, representing savings of nearly $1.2 million USD for the 4‐month study period ($3.6 million USD annually).

**Table 2:** Spending over the study period with alternative dosing strategies

Drug	Spending				
	Actual	Weight‐based (%)	Weight‐based plus vial sharing (%)	DM (%)	DM plus vial sharing (%)
Pembrolizumab	$6.53	$6.71 (+2.4%)	$5.37 (−18%)	$6.37 (−2.5%)	$5.31 (−19%)
Nivolumab	$3.30	$3.44 (+4.2%)	$3.40 (+2.9%)	$3.00 (−9.2%)	$2.99 (−9.6%)

Note

Spending is in millions of $USD

Abbreviations: %, percent change from actual spending; DM, dose minimization.

Dose minimization (the lesser of weight‐based and fixed dose) *without* vial sharing decreased nivolumab spending by 9.2%. Dose minimization *with* vial sharing decreased nivolumab spending by 9.6% (Table 2). Dose minimization *without* vial sharing decreased pembrolizumab spending by only 2.5%, but dose minimization *with* vial sharing decreased spending by 19%. Overall, dose minimization *with* vial sharing for both drugs decreased spending by 16%, representing savings of $1.5 million USD for the 4‐month study period, or $4.5 million USD annually. This corresponded to savings of more than 24 000 mg of pembrolizumab, and more than 11 000 mg of nivolumab during the 4‐month study period.

**Figure 1:**
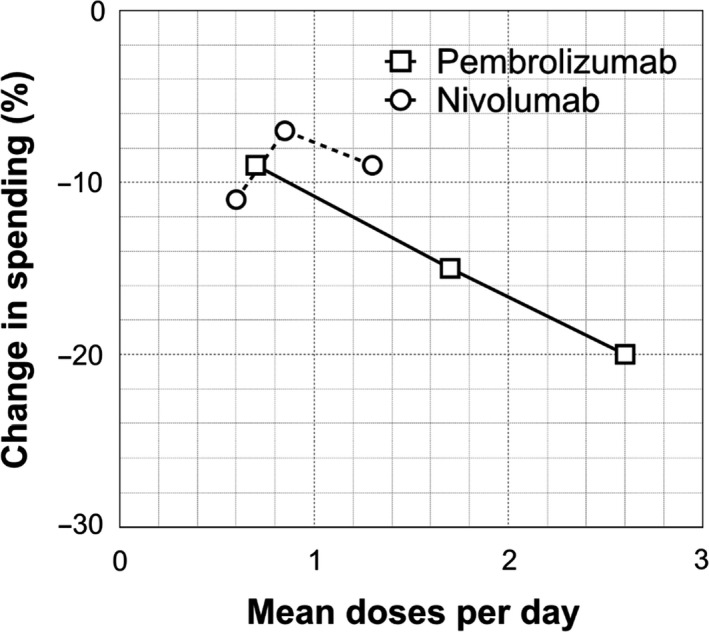
Savings from dose minimization plus vial sharing by infusion center. Change in spending = (spending from dose minimization *with* vial sharing) − (spending from current practice). Mean doses per day = mean daily doses of drug at three infusion treatment centers

The three infusion sites in the study represent a range of patient volumes, with a daily average of 45, 110, and 175 cancer patients receiving some form of treatment, a subset of whom receive pembrolizumab or nivolumab. Savings from dose minimization *with* vial sharing increased with increasing patient volume for pembrolizumab, but not for nivolumab (Figure 1). With pembrolizumab, increasing patient volume increased savings from 9% to 15% to 21%. With nivolumab, higher patient volume failed to result in greater savings.

**Figure 2:**
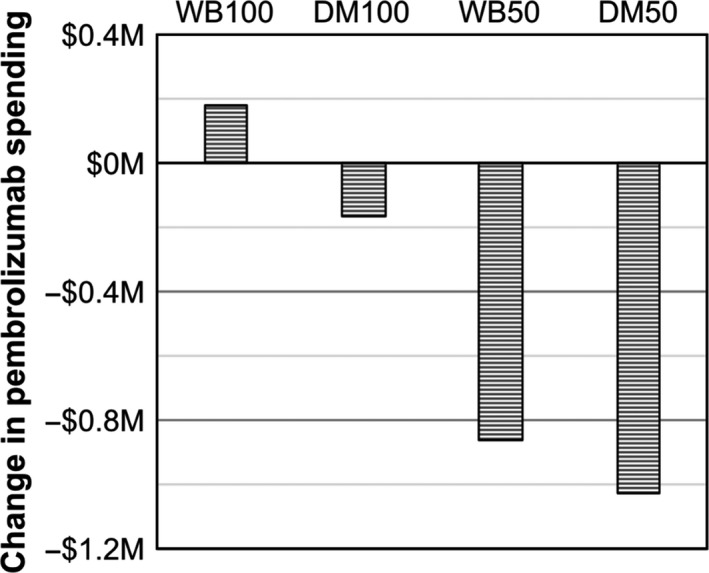
Change in pembrolizumab spending for alternative dosing strategies with 50 mg pembrolizumab vials available. Dosing strategies did not include vial sharing, and change was calculated relative to actual drug use. WB100 and WB50 = weight‐based dosing with 100 and 50 mg pembrolizumab vials. DM100 and DM50 = dose minimization with 100 and 50 mg pembrolizumab vials

We also modeled the financial impact of introducing the 50 mg pembrolizumab vial to the US as is currently available in Europe. Universal weight‐based dosing with 50 mg vials *without* vial sharing decreased pembrolizumab spending by 13%, saving $850 000 USD over the 4‐month study period (Figure 2). Dose minimization *without* vial sharing using 50 mg vials decreased pembrolizumab spending by 16%, saving $1.03 million USD over the 4‐month study period or $3.1 million USD annually.

## 4 DISCUSSION

This is the first study to our knowledge that uses real‐world, patient‐level data to show that alternate dosing strategies for pembrolizumab and nivolumab can decrease spending. Dose minimization plus vial sharing decreased total pembrolizumab and nivolumab spending by 16% relative to current practice.

The magnitude of savings for alternative dosing strategies differed for pembrolizumab and nivolumab.

For pembrolizumab, vial sharing had a major impact on savings largely by decreasing drug wastage from the single 100 mg vial size currently available in the US. For nivolumab, vial sharing had little impact on savings because it is available in three vial sizes: 40, 100, and 240 mg. Combinations of these different nivolumab vial sizes allowed for dosing to within 20 mg of the target dose‐minimization amount for all patients in this study, minimizing drug wastage even without vial sharing.

Vial size impacts wastage for many drugs. Wasted cancer medications in the US accounted for excess spending of nearly $2 billion USD in 2016. This included $200 million USD on wasted pembrolizumab alone in 2016.^21^ Bortezomib, a proteasome inhibitor used to treat multiple myeloma has been cited for wastage due to a single vial size. Although available in a 1 mg vial in some countries, bortezomib is available only in a 3.5 mg vial in the US, while the average cancer patient requires 2.5 mg based on the recommended dose of 1.3 mg/m^2^. Wasted bortezomib accounts for $309 million USD annually in the US.^21^ Pilot studies have shown that vial sharing and batching programs for bortezomib doses reduce wasted medication and pharmaceutical spending by 24%‐26%.^22^, ^23^

Average weights for American adults are 90 kg for males and 77 kg for females.^24^ Pembrolizumab conversion to a 200 mg fixed dose corresponds to an average weight of 100 kg, far exceeding population averages. Nivolumab conversion to a 240 mg fixed dose corresponds to a reasonable average body weight of 80 kg. Pharmacokinetic models have evaluated weight‐based and fixed doses for reduced variation in drug exposure. Neither dosing strategy was consistently superior across 12 antibodies.^25^ For some monoclonal antibodies, fixed doses reduce variability in drug exposure and introduce additional advantages: easier dose preparation; reduced dosing errors; and less unused "wasted" drug.^26^ Of course, these advantages require selection of the proper fixed dose.

Alternative pembrolizumab and nivolumab dosing strategies have been proposed previously. Ogungbenro et al modeled three pembrolizumab and nivolumab dosing strategies: weight‐based, fixed, and “dose banding” (weight‐based dose rounded to the nearest 10% to reduce drug wastage). Dose banding reduced pembrolizumab and nivolumab spending by 7%‐15%.^27^ The three dosing strategies generated similar area under the curve (AUC) pharmacokinetics with similar variances for nivolumab (fixed dose of 240 mg) and pembrolizumab (fixed dose of 150 mg). However, both AUC and variance were significantly higher for pembrolizumab with the FDA‐approved fixed dose of 200 mg.

Data indicate that increased pembrolizumab and nivolumab exposure does not improve efficacy. A randomized dose comparison for pembrolizumab in advanced melanoma compared the weight‐based dose of 2 mg/kg to the fivefold higher weight‐based dose of 10 mg/kg. Overall response rates were an identical 26% for both doses.^4^ Thus, efficacy would not increase with a mere 1.33‐fold increase in drug exposure using a fixed dose of 200 mg rather than the appropriate 150 mg dose. Weight‐based doses of nivolumab as low as 0.3 mg/kg have been shown to saturate peripheral PD‐1 receptors, and the exposure‐response curve flattens between 1 and 3 mg/kg, suggesting no increase in efficacy beyond this dose range.^28^, ^29^ Increased exposure without therapeutic benefit is “wasted” drug spending.

Unnecessarily high drug exposures raise safety concerns, although data from randomized clinical trials for pembrolizumab and nivolumab seem to suggest that increased doses do not increase toxicity. For example, the fivefold higher dose of pembrolizumab in the melanoma trial did not increase the incidence of autoimmune reactions or other identified toxicities.^4^ However, it is possible that higher doses of PD‐1 antibodies could lead to cross‐reactivity toward unintended antigens. For example, PD‐1 antibodies cross react in a low affinity, dose‐dependent manner with nuclear antigens in dying murine cells^30^ and a human pituitary antigen.^31^ The clinical significance of these observations is currently unclear, but given the wide variety of polymorphisms seen in the general population, it is possible that low frequency, dose‐dependent, off‐target effects could occur and would likely not be detected in randomized clinical trials. PD‐1 antibodies are also immunogenic. Treatment with immune checkpoint inhibitors can result in the development of antidrug antibodies, including antibodies with the potential to neutralize the binding of the checkpoint inhibitor to its ligand although they are currently of unclear clinical significance.^32^

Several caveats to the strategies of vial sharing and dose minimization require examination. Cost analyses did not consider the increased drug preparation time (estimated at 3‐5 minutes per patient dose) in our pharmacy. The calculations restricted vial sharing to patients treated on the same day at the same site, but did not account for a small number of cases that would violate our current pharmacy policy of discarding opened unused vials after 6 hours. Drugs held in reserve for vial sharing with another patient complicate pharmacy workflow. Dose minimization requires calculations that could lead to errors. Repeated access of single vials to treat multiple patients has been linked to infectious complications.^33^, ^34^ Finally, as the distribution of patient weights and availability of other pembrolizumab and nivolumab vial sizes may be different around the world, the magnitude of savings with novel dosing strategies may be different in other countries.

Vial sharing by splitting 100 mg vials into two 50 mg aliquots with subsequent administration of a 150 mg fixed dose would decrease dose preparation time, eliminate dose calculation, and simplify pharmacy workflow, mitigating the caveats. Furthermore, most of the benefits from vial sharing would be achieved if the 50 mg vials available elsewhere become available in the US. Implementation of a 150 mg fixed dose at our center would have saved more than $4 million USD over 1 year. The potential savings on a national level are enormous.

A barrier to achieving the savings identified in this study may be reluctance of providers to deviate from the current FDA‐approved fixed doses. In the absence of clinical evidence for superiority, regulatory bodies should eliminate inappropriate fixed doses and allow both weight‐based and appropriate fixed doses. The pharmaceutical industry should be compelled to provide appropriate vial sizes. Compensation to prescribers should be limited to the lowest dose required for full therapeutic effect.

## 5 CONCLUSIONS

In the setting of equivalent clinical efficacy and safety, economic considerations should be an important factor in clinical decision‐making. By making minor changes in the dosing of pembrolizumab and nivolumab, our academic medical center alone would have created savings of more than $4.5 million USD annually. Based on worldwide usage of pembrolizumab and nivolumab exceeding $14 billion USD annually, adoption of dose minimization plus vial sharing could reduce spending by more than $1 billion USD annually if similar savings to those found in our study were achieved worldwide. Clinicians can implement the strategies proposed here immediately without any action from pharmaceutical companies, payers, regulatory agencies, or other parties. Physicians and pharmacists have no control over many aspects of medical economics, but they can adopt an appropriate dosing strategy to achieve major savings.

## CONFLICTS OF INTEREST

Dr Hall has previously received institutional research support from Noona Healthcare (now Varian) and personal fees from Cancer Support Community, both outside the submitted work. Dr Zhang has nothing to disclose. Dr Kim has received speaker bureau fees from Bristol‐Myers Squibb. Ms Hwang has nothing to disclose. Dr Chu has nothing to disclose. Dr Bhatia has received personal fees from Bristol‐Myers Squibb, personal fees from EMD‐Serono, personal fees and other from Sanofi‐Genzyme, grants from Bristol‐Myers Squibb, grants from EMD‐Serono, grants from Merck, grants from NantKwest, grants from Novartis, grants from Immune Design, grants from Oncosec, grants from Exicure, grants from Nektar, all of which are outside the submitted work. Dr Reddy has nothing to disclose.

## AUTHORS’ CONTRIBUTION

EH contributed to the conceptualization, data curation, formal analysis, investigation, methodology, supervision, visualization, writing—original draft, and writing—review and editing. JZ contributed to the conceptualization, data curation, formal analysis, writing—original draft, and writing—review and editing. EJK contributed to the conceptualization, formal analysis, and writing—review and editing. GH contributed to data curation, formal analysis, and writing—review and editing. GC contributed to methodology, visualization, supervision, and writing—review and editing. SB contributed to conceptualization, formal analysis, supervision, and writing—review and editing. SR contributed to conceptualization, data curation, supervision, and writing—review and editing. All authors approve the submitted version of this manuscript.

## Data Availability

Research data are not shared as this relates to proprietary information regarding infusions and patient volumes of our academic cancer center.
